# Oxidative Stress‐Related Programmed Cell Death in Male Infertility: Focussing on Ferroptosis

**DOI:** 10.1111/jcmm.71002

**Published:** 2026-05-24

**Authors:** Nafiseh Sanei‐Ataabadi, Fatemeh Aboutalebi, Kianoush Dormiani, Mohammad Hossein Nasr‐Esfahani

**Affiliations:** ^1^ Department of Biology Naghshejahan Higher Education Institute Isfahan Iran; ^2^ Department of Animal Biotechnology, Cell Science Research Center Royan Institute for Biotechnology, ACECR Isfahan Iran; ^3^ Department of Animal Biotechnology, Reproductive Biomedicine Research Center Royan Institute for Biotechnology, ACECR Isfahan Iran

**Keywords:** apoptosis, autophagy, ferroptosis, male infertility, oxidative stress, testicular cell

## Abstract

Oxidative stress is a common occurrence in testicular cells of infertile men and is considered a significant cause of male reproductive dysfunction. The increase in reactive oxygen species (ROS) leads to impaired spermatogenesis by activating caspases and generating free radicals from mitochondria, resulting in DNA damage and cell death through apoptosis. Additionally, oxidative stress in the testis triggers autophagy as an alternative form of programmed cell death in male germ cells. There is a complex interplay between autophagy and apoptosis in response to oxidative stress in male germ cells. Recent studies have shown that oxidative stress can also induce a form of cell death called ferroptosis, which is an iron‐regulated, caspase‐independent pathway. Ferroptosis is initiated by the inactivation of the antioxidant enzyme glutathione peroxidase 4 (GPX4) and the dysfunction of system Xc‐, leading to iron‐dependent lipid peroxidation. Therefore, a combination of apoptosis, autophagy and ferroptosis determines the fate of testicular cells under oxidative stress conditions. This review explored these three types of programmed cell death associated with oxidative stress in testicular cells and their implications for male infertility, with a focus on ferroptosis.

## 1 Introduction

Infertility is a global reproductive health problem defined as the failure to conceive after 1 year of unprotected sexual intercourse, affecting 13%–15% of couples worldwide [1]. Male factor infertility is responsible for approximately half of infertility cases. Multiple factors contribute to the pathogenesis of male infertility, including the excessive production of reactive oxygen species (ROS) and subsequent oxidative stress. Oxidative stress occurs when the production of ROS exceeds the body's antioxidant defences [2, 3]. ROS production is a normal process in the male germ line and plays a crucial role in physiological pathways. However, when ROS production becomes excessive and overwhelms the body's antioxidant systems, it can negatively impact male fertility and embryonic development [4].

Sperm cells are particularly vulnerable to oxidative damage, owing to the abundance of unsaturated fatty acids in their cytoplasmic membranes. This susceptibility is exacerbated by their limited ability to repair damage caused by oxidative stress, as they lack essential cytoplasmic enzymes. Consequently, oxidative stress can lead to lipid peroxidation, ATP depletion and reduced sperm motility and ultimately cell death [5]. Oxidative stress can contribute to male infertility in two main ways. Firstly, during the final stage of differentiation from round spermatid to sperm and throughout sperm maturation, it leads to the production of sperm with low motility, abnormal morphology, high DNA fragmentation and an inability to perform essential physiological functions such as capacitation, acrosome reaction, binding to zona pellucida, fertilisation and supporting development to term. Secondly, it can reduce testicular output, known as oligozoospermia, during spermatogonia proliferation and meiotic division [6].

Understanding the cellular mechanisms involved in cell death is crucial for comprehending how reactive oxygen species (ROS) impact mitotic and meiotic cell division in the testis. When cells are under oxidative stress, they can activate apoptosis as a defence mechanism. This process is mediated by ROS, which triggers a cascade involving cytochrome c, caspase‐3 and caspase‐9, ultimately leading to significant DNA fragmentation in both single and double strands [7, 8]. Additionally, autophagy, a self‐cleaning process also known as programmed cell death II, is activated during stressful conditions like oxidative stress to break down and recycle damaged components within cells [9]. Although it was believed that oxidative stress in male germ cells primarily induces apoptosis and autophagy as the main types of programmed cell death, recent evidence has demonstrated the involvement of a caspase‐independent form of cell death known as ferroptosis following oxidative stress [10]. Ferroptotic cell death is characterised by three main hallmarks: a reduction in the antioxidant glutathione (GSH) and a decrease in glutathione peroxidase 4 (GPX4) activity, an accumulation of lipid peroxidation products, resulting in cell membrane perturbation and an elevation of free iron levels, leading to the ROS generation through the Fenton reaction [11, 12]. Recent studies suggest that ferroptosis plays a significant role in the pathophysiology of the testis through all these hallmarks [13].

In this review, we discuss the sources of ROS in male germ cells and the major programmed cell death forms associated with oxidative stress, including apoptosis, autophagy and ferroptosis with a focus on male infertility.

## 2 ROS in Male Germ Cells

ROS include highly reactive free radicals, including superoxide anions (O_2_•^−^), hydrogen peroxide (H_2_O_2_), peroxyl (ROO•) and hydroxyl (OH•) [14]. In the male reproductive system, sources of ROS production can be classified into endogenous and exogenous factors. The most critical endogenous sources of ROS production are leukocytes and immature spermatozoa [15]. Leukocytes, which produce significantly more ROS than normal sperm cells, are responsible for oxidative stress in seminal fluid. Granulocytes and macrophages are the predominant cell types found in semen and contribute to ROS generation. This process is mainly linked to glucose‐6‐phosphate dehydrogenase (G6PDH) activity, which produces high NADPH levels. NADPH, in turn, strongly stimulates NADPH oxidase, a major ROS producer. Immature spermatozoa also contribute to ROS production due to their incomplete maturation process. Furthermore, Sertoli cells, which play a crucial role in spermatogenesis, have been scientifically recorded to produce ROS, which can be hindered by scavengers [15]. Another possible contributing endogenous factor to oxidative stress in male germ cells is varicocele. Varicocele, the predominant cause of male infertility, has been associated with elevated oxidative stress, sperm DNA damage‐induced ROS and heightened scrotal temperature. These observations have been supported by data illustrating decreased levels of seminal lipid peroxidation and sperm DNA damage following varicocelectomy [16].

**FIGURE 1:**
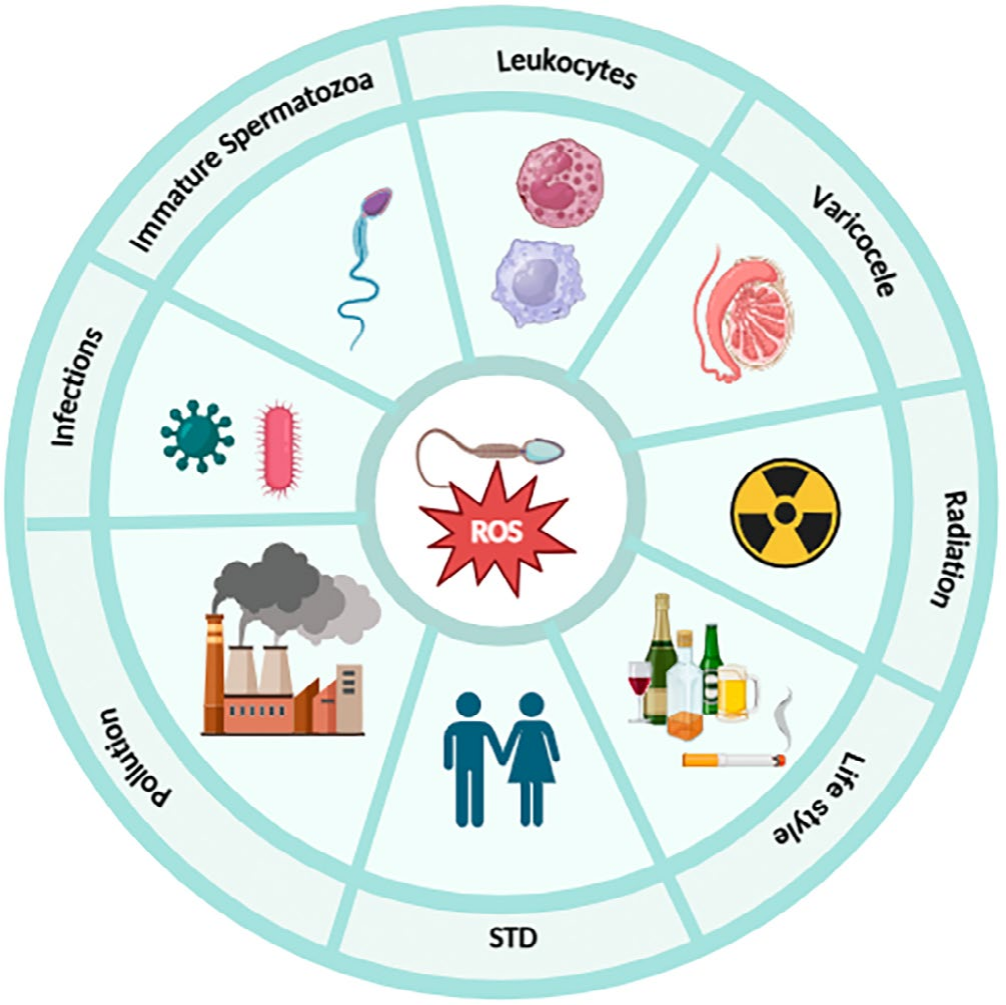
The most critical sources of ROS in male germ cells. Immature spermatozoa, leukocytes and varicocele are the most pivotal endogenous sources of ROS. Moreover, environmental pollution, radiation, lifestyle choices such as smoking and alcohol consumption, bacterial/viral infections and sexually transmitted diseases (STDs) are the primary exogenous sources of ROS.

Exogenous factors that can exacerbate ROS production and inflammation include environmental pollution, radiation, lifestyle choices such as smoking and alcohol consumption, bacterial/viral infections, microorganism mutations, or sexually transmitted diseases (Figure 1) [17, 18]. In addition, certain toxins, such as cadmium, can cause mitochondrial dysfunction, elevate mitochondrial superoxide and cellular ROS levels, trigger excessive mitochondrial fission and release cytochrome c, leading to apoptosis [19].

ROS impacts the germ cells during different stages of development, including primary and secondary spermatocytes and spermatozoa. However, among the various cell types, sperm is particularly vulnerable to damage caused by ROS, and this decline in sperm quality is evident in the offspring [4]. Numerous studies have demonstrated that low levels of ROS promote sperm capacitation, hyperactivation, acrosome reaction, motility and chromatin condensation in maturing spermatozoa [20, 21]. Additionally, ROS enhances sperm binding to the zona pellucida, facilitating sperm‐oocyte fusion [21]. However, excessive production of ROS has harmful effects on male germ cells by damaging DNA, lipids and proteins [16, 22]. Excessive levels of ROS in semen have been observed in a significant portion of infertile men. High ROS levels are negatively correlated with key sperm quality parameters, including concentration, motility and morphology, indicating that excessive oxidative stress plays a major role in male infertility [23].

## 3 Apoptosis in Male Infertility

In the mammalian testis, germ cells undergo several rounds of mitotic division before progressing to meiotic division, ultimately leading to the formation of mature spermatozoa [24]. However, under physiological conditions, approximately 50%–70% of germ cells undergo apoptosis during spermatogenesis in seminiferous tubules [25]. This process helps maintain a balance between the number of germ cells and the supportive capacity of Sertoli cells [25]. Apoptosis in the testis also plays an important role in eliminating defective germ cells and ensuring quality control in sperm production. Male germ cell apoptosis involves two main pathways, the extrinsic and intrinsic pathways, which operate independently but are interconnected and mutually influence each other [26].

### 3.1 The Extrinsic Apoptotic Pathway in Male Germ Cells

The extrinsic or death receptor‐initiated pathway involves the activation of Fas transmembrane death receptors as a member of TNF families, mediating the FasL ligand. Fas (CD95/APO‐1) and FasL (CD95L), two pro‐apoptotic transmembrane proteins, belong to the TNF receptor and the TNF superfamily, respectively [27]. Although the Fas receptor is expressed in a wide range of tissues, the expression of FasL is restricted to some immune system cell types and testicular Sertoli cells [28]. In the mammalian testis, FasL expressed from Sertoli cells interacts with the Fas receptor on the germ cell surface to initiate the death receptor apoptotic pathway [25]. This pathway is continued by the trimerisation of Fas and recruitment of the cytoplasmic adapter protein called Fas‐associated death domain (FADD), which, in turn, recruits Caspase‐8 to form the complex called DISK (Death‐inducing signalling complex). The caspase 8/DISC complex activates effector Caspase‐3, ultimately increasing poly‐(ADP)‐ribose polymerase (PARP) cleavage, DNA fragmentation and cell death [29].

### 3.2 The Intrinsic Apoptotic Pathway in Male Germ Cells

The intrinsic or mitochondria‐dependent pathway is another crucial apoptotic pathway in male germ cells. The mitochondrial pathway is mainly regulated by Bcl‐2 family proteins, including the anti‐apoptotic proteins (Bcl‐2 and Bcl‐xL) and pro‐apoptotic proteins (Bax and Bak). Under normal conditions, anti‐apoptotic proteins form heterodimers and protect mitochondrial outer membrane integrity. However, under stress conditions, such as hypoxia, oxidative stress and infection, pro‐apoptotic proteins are translocated to the mitochondria to form a heterodimer with anti‐apoptotic proteins, causing mitochondrial outer membrane permeabilisation that results in the release of mitochondrial components into the cytoplasm [30]. One such component is cytochrome c, which interacts with the apoptotic protease activating factor‐1 (Apaf‐1) leading to the assembly of the apoptosome. The apoptosome binds to procaspase‐9 and activates caspase‐9, resulting in the proteolytic activation of caspase‐3. This leads to poly‐(ADP)‐ribose polymerase (PARP) cleavage, DNA fragmentation and ultimately cell death [31]. Despite the dependency of the intrinsic and extrinsic pathways of male germ cell apoptosis, cross‐talks occur between them. Activated caspase‐8 in the extrinsic pathway results in the cleavage of a member of the Bcl‐2 family named Bid. The cleaved Bid or tBid can activate Bak and Bax after translocation to the mitochondria, promoting cytochrome c release [32].

### 3.3 Relationship Between Oxidative Stress and Apoptosis in Male Infertility

The majority of research indicated high rates of apoptosis in the testis of infertile men. Among the various inducers of apoptosis in the testis, oxidative stress plays a significant role. Previous studies have shown a strong correlation between ROS levels and apoptosis rates in ejaculated semen specimens from men with idiopathic infertility. Furthermore, there is a positive association between increased sperm damage caused by ROS and the upregulation of proteins such as caspase‐3, caspase‐9 and cytochrome c in these patients [33]. Studies using rat testicular ischemia–reperfusion injury (tIRI) models have shown that increased inflammatory cytokines (e.g., INF1α, IL‐1β) activate the JNK signalling pathway, leading to endothelial activation and leukocyte infiltration, which elevates ROS production [34]. In response to oxidative stress in male germ cells, Thioredoxin (Trx) acts as an inhibitor of apoptosis signal‐regulating kinase 1 (ASK‐1). When oxidised, Trx dissociates from ASK‐1 and activates the ASK1 signalosome. ASK1, as a member of the mitogen‐activated protein kinase (MAPK) family, triggers the c‐Jun N‐terminal kinase (JNK) and p38 MAPK pathways, leading to the activation of the pro‐apoptotic Bax protein and the mitochondrial pathway [35]. Moreover JNK promotes hydrogen peroxide–induced apoptosis in germ cells by elevating ROS levels, reducing the activities of antioxidant enzymes such as superoxide dismutase (SOD) and catalase and increasing the accumulation of the oxidative stress marker MDA [34]. An alternative pathway contributing to JNK‐mediated apoptosis involves JNK phosphorylating the p53 family of proteins, which prevents their ubiquitin‐mediated degradation, thereby stabilising p53 levels. This stabilisation leads to the upregulation of pro‐apoptotic genes such as Bax and PUMA (p53 up‐regulated modulator of apoptosis), promoting apoptosis [36]. Excessive ROS triggers the disruption of the mitochondrial membrane by oxidising cardiolipin (a phospholipid in the inner mitochondrial membrane), depolarising the mitochondrial membrane and activating the Bax/Bak channels. These events result in the release of cytochrome c from the mitochondria into the cytoplasm, leading to the formation of the apoptosome and activation of caspases [8]. The release of cytochrome c also exacerbates ROS production and DNA damage, further enhancing the apoptotic pathway [21].

**FIGURE 2:**
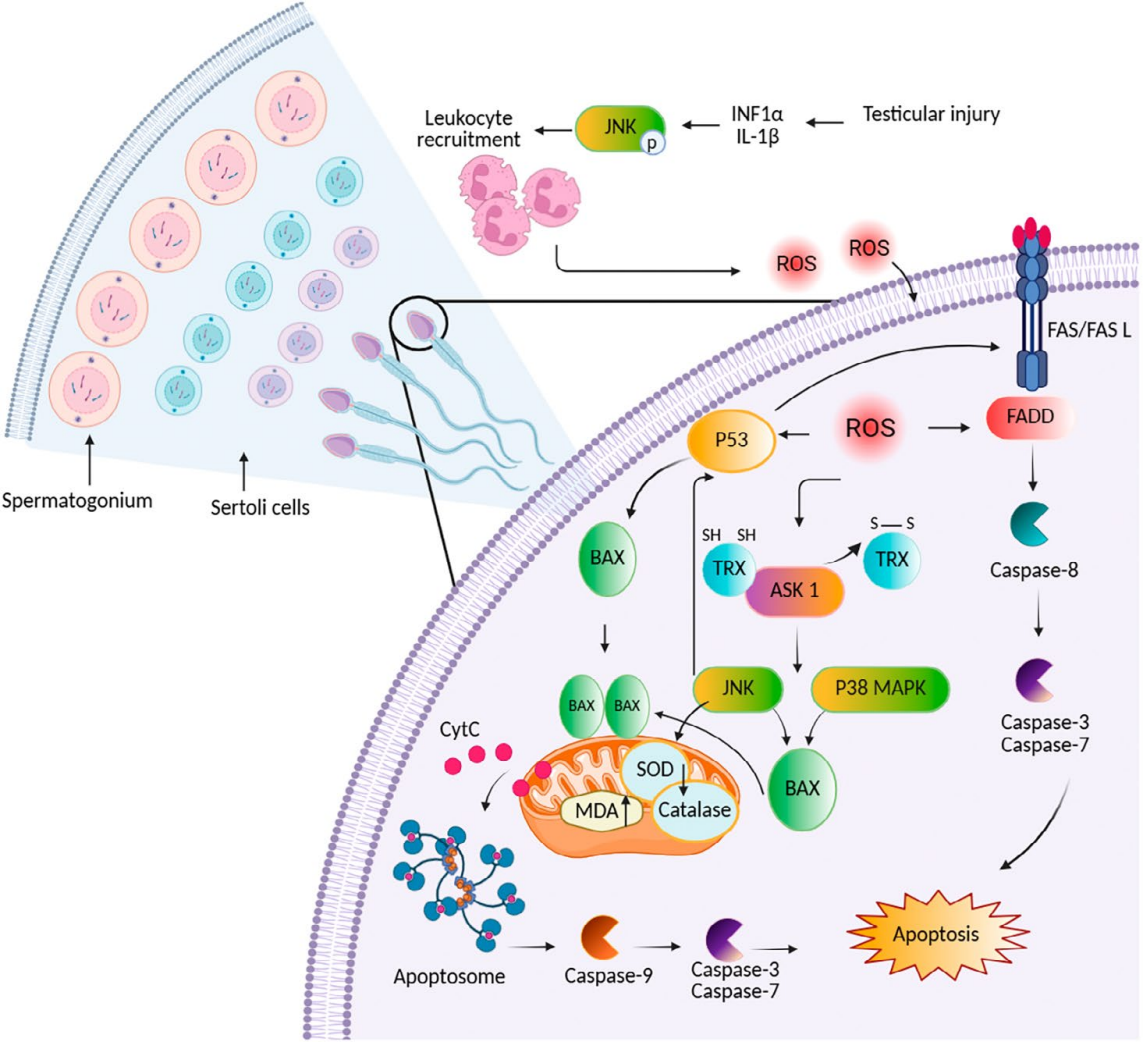
Oxidative stress induces apoptosis in male germ cells through intrinsic and extrinsic pathways. Testicular ischemia–reperfusion injury triggers endothelial activation and elevated proinflammatory cytokines (INF1α, IL‐1β), which phosphorylate JNK and promote leukocyte infiltration. The resulting increase in ROS activates the mitochondrial apoptotic pathway. In this pathway, an excess of ROS disrupts the mitochondrial membrane, releasing cytochrome c into the cytoplasm. Cytochrome c binds with Apaf‐1, triggering the apoptosome formation and activating caspase‐9. This activation of caspase‐9 initiates the caspase cascade, ultimately leading to apoptosis. Additionally, ROS can activate Trx, leading to its oxidation, detachment from ASK‐1 and subsequent activation of the ASK1 signalosome. ASK1 then triggers the JNK and p38 MAPK pathways, activating the pro‐apoptotic Bax protein and the subsequent mitochondrial pathway. Simultaneously, JNK in germ cells increases ROS and MDA levels while reducing the activities of antioxidant enzymes SOD and CAT. In addition, JNK can promote apoptosis by phosphorylating p53 proteins, which prevents their degradation and stabilises p53 levels. In the extrinsic pathway, ROS accumulation can activate the Fas receptor and FasL ligand, resulting in DISC complex formation, which further activates the caspase cascade. Both the intrinsic and extrinsic pathways converge on caspase 3, ultimately leading to DNA fragmentation and apoptosis.

Excessive production of ROS has been reported to induce apoptosis in male germ cells through the Fas/FasL as well as mitochondrial pathways. ROS accumulation can activate the Fas receptor and FasL ligand, leading to the formation of the DISC complex and activation of caspase‐8 [37]. An analysis of apoptotic signalling pathways in male germ cells revealed an increase in the expression of extrinsic markers (Fas, FasL and caspase‐8) and intrinsic markers (Bid, Bak, Bad, Bax and caspase‐9) along with p53. Simultaneously, there was a decrease in the Bcl‐2 expression at high concentrations of H_2_O_2_ exposure. Excessive ROS can damage sperm cells by affecting their genetic material in the absence of strong antioxidant protection. High levels of single/double‐strand DNA breaks induced by ROS, as indicated by 8‐hydroxy‐2′‐deoxyguanosine (8OHdG) adducts, are a critical hallmark of ROS‐induced DNA damage that activates the apoptotic pathway [33]. In addition to single/double‐strand DNA breaks, oxidative stress generates other forms of DNA damage in spermatozoa including purine, pyrimidine and deoxyribose modifications, the introduction of abasic sites and DNA cross‐linking. These damages can disrupt gene transcription, decrease telomeric DNA length and induce GC to TA transversion [38]. In response to oxidative stress, activated p53 acts as a guardian of the genome, regulating the quality of male germ cells by inducing spontaneous apoptosis in DNA‐damaged conditions [39]. In cases of severe DNA damage that threatens genome integrity and male germ cell quality, p53 upregulates the expression of the pro‐apoptotic BAX protein or downregulates the anti‐apoptotic Bcl‐2 protein, which promotes apoptosis through the intrinsic mitochondrial pathway [31]. Additionally, p53 activates the Fas‐extrinsic apoptotic pathway, establishing a crosstalk between these two pathways [32] (Figure 2). Another pathway for oxidative damage in male germ cells is the oxidation of polyunsaturated fatty acids (PUFAs) that are abundant in their plasma membrane. Peroxidation of these lipids by ROS results in the production of highly mutagenic and genotoxic electrophilic aldehydes such as malondialdehyde (MDA) and 4‐hydroxynonenal (4‐HNE) [40, 41]. There is a negative correlation between seminal MDA concentrations with sperm motility and concentration [41]. Similarly, 4‐HNE can negatively impact sperm motility, morphology, acrosomal reaction and interactions with the zona pellucida of oocytes [42, 43, 44]. These byproducts can interact with essential macromolecules such as proteins, nucleic acids and membrane phospholipids. The interaction of lipid aldehydes with electron transport chain proteins in mitochondria results in the transfer of electrons from these proteins to the final electron acceptor, oxygen, generating more radical O_2_^−^ [44, 45]. This process ultimately enhances lipid peroxidation, mitochondrial ROS production, DNA fragmentation and apoptosis [46, 47]. Apoptosis of seminiferous germ cells is a significant factor affecting sperm quality and is considered a key contributor to male infertility in varicocele [46, 47]. Numerous studies have shown an increase in apoptotic markers including externalisation of phosphatidylserine, nuclear DNA fragmentation, mitochondrial dysfunction and altered expression of FasL, Bax and caspases in infertile men with varicocele. These changes are primarily attributed to heat stress and excessive production of ROS [46, 48, 49, 50, 51].

## 4 Autophagy in Male Infertility

### 4.1 Autophagy Definition

Autophagy, also known as programmed cell death II, eliminates unnecessary cytoplasmic components, including damaged organelles and protein aggregates through lysosomal digestion. While traditionally viewed as a survival mechanism, recent research suggests that autophagy can also induce cell death [7]. Autophagy plays a critical role in sperm production, and its deficiency can give rise to male infertility. It is essential for maintaining spermatogonia stem cells (SSCs) and supporting the survival of differentiated spermatogonia [52, 53]. Autophagic cell death is distinct from apoptotic cell death in three main ways: (1) the presence of numerous autophagic structures within the dying cell, (2) the lack of phagocytes engulfing the dying cell and (3) the potential independence from caspase activation in some cases [7, 53]. The ULK1 complex is the main initiator of autophagy, and its activity is regulated by the mTOR pathway. Under normal conditions, mTOR is active and inhibits ULK1 complex formation [54]. Various stimuli can trigger autophagy, such as mitochondrial dysfunction, amino acid deprivation, hypoxia, unfolded protein accumulation and oxidative stress. This process is regulated by more than 30 Autophagy‐related (Atg) genes in yeast [55, 56]. Upon stimulation, mTORC1 (mammalian target of rapamycin) is inhibited, leading to the activation of the Unc51‐like Autophagy Activating Kinase 1 (ULK1) complex [57]. The ULK1 complex then translocates PI3K (phosphatidylinositol 3‐kinase) to the endoplasmic reticulum. The PI3K complex, consisting of class III PI3K, ATG14L, Beclin‐1 and p150, is necessary for vesicle nucleation to initiate autophagosome assembly [8, 58]. Multiple proteins, such as Beclin‐1 and members of the autophagy‐related gene (ATG) family, are essential for autophagy, contributing to phagophore formation and the recruitment of autophagy‐associated proteins. Additionally, microtubule‐associated protein light chain 3 (LC3) plays a key role in phagophore elongation, closure and autophagosome maturation [54, 59]. After the formation of the double‐membrane autophagosome that encapsulates the targeted substrates, the autophagosome's outer membrane fuses with the lysosomal membrane, generating an autolysosome. Within the autolysosome, the contents undergo degradation and are subsequently recycled by the cell [60].

### 4.2 The Role of Autophagy in Male Germ Cells

An increasing body of research indicates that autophagy plays a pivotal role in male reproduction [61]. For instance, autophagy is significant in the regulation of spermatogenesis, providing cytoprotective function during the differentiation of spermatogonia into spermatozoa and in the survival of differentiated spermatogonia, as well as in maintaining SSCs [52, 62]. Testosterone production is crucial for male development and the maintenance of sexual function, primarily occurring in Leydig cells, where there is a notable level of autophagic activity. Autophagy is involved in testosterone production, and dysfunction in autophagy may contribute to a decline in testosterone production [61, 63].

Previous research has highlighted the importance of autophagy in acrosome development. A study by Wang et al. revealed that mice with a germ cell‐specific deletion of Atg7 were unable to reproduce due to impaired acrosome biogenesis, resembling human globozoospermia [64]. Moderate autophagy helps maintain organism homeostasis and protects against testicular damage induced by hyperglycemia and hypoxia. However, dysregulated autophagy has been linked to male infertility in several research studies [65]. Liu et al. demonstrated that impaired autophagy, particularly in Sertoli cells, impacts the reproductive capacity of male mice. This impact results from irregular seminiferous tubules arrangement and sperm cells with abnormal head shapes. Autophagy‐deficient mice showed disruptions in cytoskeletal element organisation with an accumulation of PDLIM1 as a negative regulator of cytoskeleton organisation within the testis [66].

### 4.3 Critical Regulators of Autophagy in Male Germ Cells

In mammalian cells, mTOR is a crucial regulator of autophagy, suppressing autophagy under normal conditions. However, after serum starvation or environmental stress, mTOR is inactivated, triggering autophagy. Other critical regulators of autophagy include PI3Ks, MAPK and HIF‐1α/beclin‐1 signalling pathways [30, 67]. Aparicio et al. evaluated autophagy and mitophagy, the process of mitochondria degradation via autophagy in human spermatozoa. They reported that LC3, Atg5, Atg16, Beclin 1, p62, mTOR, AMPKα 1/2 and PINK1 are present and functionally active in human spermatozoa. The study concluded that mitophagy, a specific form of autophagy, may regulate the motility and viability of human spermatozoa [68]. Furthermore, Atg7 is believed to play a crucial role in acrosome biogenesis, an essential organelle in the fertilisation process such that knockout of the Atg7 gene in mice leads to infertility with characteristics similar to human globozoospermia [64].

### 4.4 Relationship Between Oxidative Stress and Autophagy

Excessive ROS buildup causes oxidative stress and cellular damage, triggering autophagy as a protective mechanism to remove damaged organelles and restore balance. Through mitophagy and pexophagy, cells selectively degrade defective mitochondria and peroxisomes to limit ROS production. However, prolonged oxidative stress can hinder autophagy, reducing the clearance of protein aggregates and further increasing ROS accumulation [69]. ROS activates cellular autophagy by triggering various downstream signalling pathways. ROS can affect the AMP‐activated protein kinase (AMPK) through direct and indirect mechanisms, resulting in implications for the initiation of autophagy. AMPK activation causes ULK1 phosphorylation and inhibition of TORC1, which in turn stimulates autophagy [70]. Furthermore, ROS induces autophagy by negatively regulating the PI3K/Akt/mTOR signalling pathway in mammalian germ cells [65].

**FIGURE 3:**
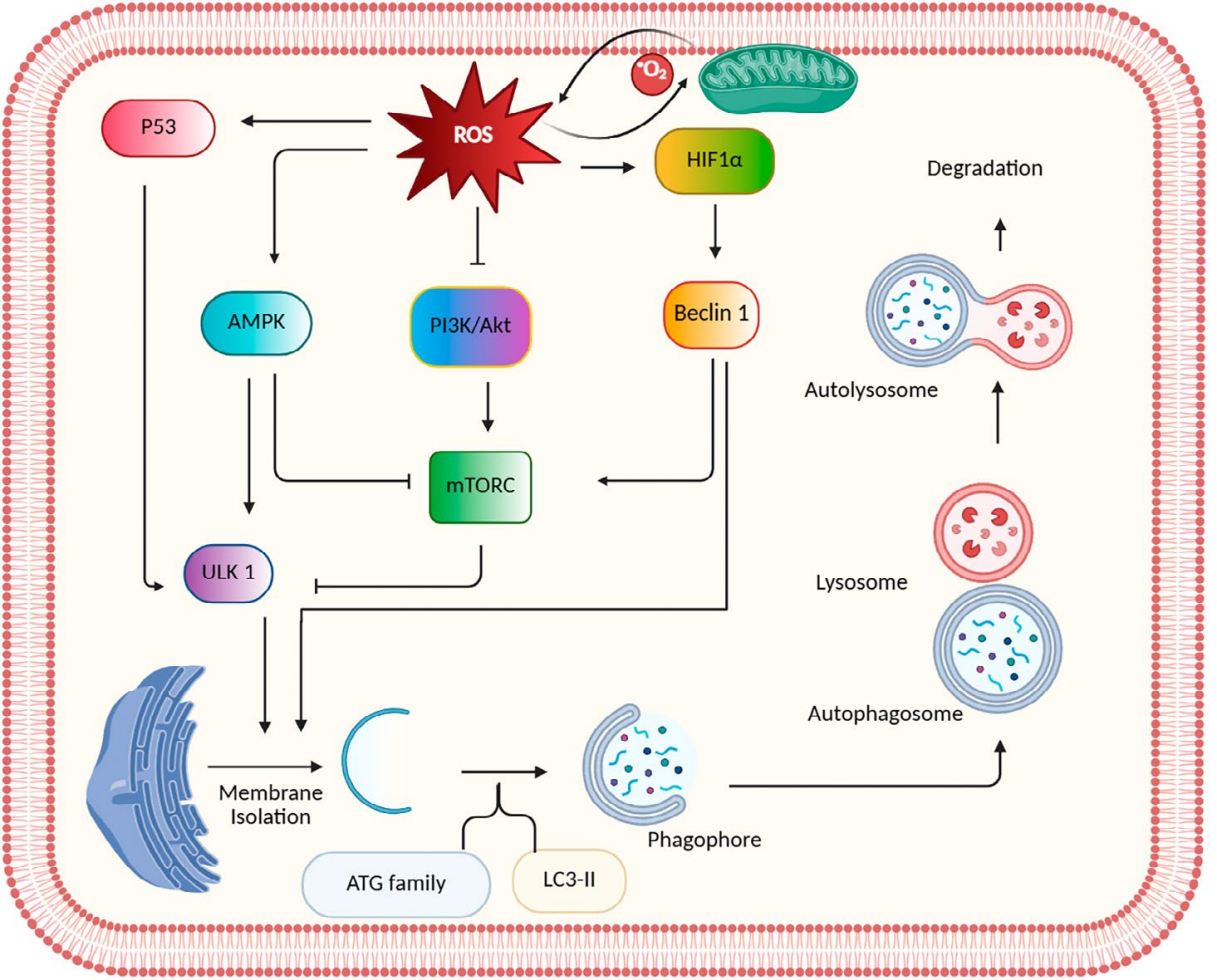
An overview of the relationship between oxidative stress and autophagy in mammalian germ cells. Autophagy begins with the formation of a phagophore, a double membrane that engulfs cellular components for degradation. The process is controlled by the ULK1 complex, which is regulated by the mTOR pathway. In the normal condition, mTOR inhibits ULK1 and suppresses autophagy, but under prolonged oxidative stress, mTOR inhibition mediated by PI3K/Akt and AMPK activates ULK1, initiating the process. The mitochondrial electron‐transport chain is identified as the primary endogenous source of H_2_O_2_ and O_2_•− for initiating autophagy in testicular cells. Furthermore, an increase in ROS generation activates transcription factors like HIF‐1α, which ultimately enhances the expression of Beclin 1. The phagophore expands into an autophagosome that fuses with lysosomes to form an autolysosome for degradation. Key proteins such as ATGs, Beclin‐1 and LC3 coordinate the different stages of autophagy.

In the realm of male reproduction, the pivotal involvement of mitochondria is observed in the process of germ cell development, wherein any abnormalities in mitochondrial quality may result in significant implications for male fertility. The association between mitochondrial dysfunction and oxidative stress is well‐established, given that mitochondria are both recipients and sources of ROS within spermatogenic cells [71]. Studies have indicated that the generation of ROS in mitochondria is crucial for regulating autophagy [72]. Various scientific investigations assert that the mitochondrial electron‐transport chain serves as the principal endogenous origin of H_2_O_2_ and O_2_•− for facilitating autophagy initiation in testicular cells. The proteins within mitochondria are altered by H_2_O_2_, impairing the electron transfer mechanism and generating intracellular O2•− [73]. Moreover, an escalation in ROS production instigates the activation of transcription factors such as HIF‐1α, AP‐1, NF‐κB, p53, FOXO3 and Nrf2. The occurrence of several autophagy mechanisms depends on the severity and duration of oxygen deficiency due to hypoxia. Moderate and persistent hypoxia prompts the activation of HIF‐1α and PKC‐JNK1‐mediated pathways to initiate autophagy. In response to the production of ROS, HIF‐1α is mobilised to mitochondria. When exposed to mild hypoxia, ROS can trigger BNIP3 and avert the inhibition of Beclin‐1 by Bcl‐2, consequently promoting the release of Beclin‐1 and inducing autophagy [8]. Oxidative stress activates p53, which in turn induces autophagy through the transcription of several autophagy‐related genes, such as Ulk1 and Atg7. However, there is an interesting connection between autophagy and p53. Autophagy inhibits p53, while p53, in turn, stimulates autophagy (Figure 3) [74].

Glutathione (GSH) plays a crucial role in the antioxidant defence of spermatogonial cells. Depletion of GSH triggers autophagy by downregulating S‐glutathionylated proteins [75]. Autophagy initiation can be triggered by GSH depletion through a signalling pathway that is independent of AMPK [76]. Zhang et al. demonstrated that hyperthermia induces autophagy features in germ cells, such as autophagosome formation and LC3‐I to LC3‐II conversion in germ cells. They also highlighted that autophagy has a potential role in germ cell death and is associated with apoptosis in germ cells after heat treatment [55]. In another study, Zhu et al. demonstrated that hypoxia‐induced by varicocele leads to autophagy through the HIF1α/BNIP3/Beclin1 signalling pathway in the testis of rats with varicocele. The autophagic process, in conjunction with apoptosis, may be essential in male infertility associated with varicocele [38]. Our recent study supported the up‐regulation of autophagy in the testes and sperm of rats with varicocele, showing increased expression of Atg7 protein content and the LC3‐II/LC3‐I protein ratio in the varicocele group compared to the control [77]. Recent research has indicated that autophagy can be induced by endogenous ROS and exogenous‐derived ROS. For instance, excessive copper in the form of copper sulfate (CuSO4) can promote autophagy in the testes and mouse‐derived spermatogonia cells through the AMPK‐mTOR pathway [78]. Xenobiotics, particularly endocrine‐disrupting chemicals (EDCs), wreak havoc on reproductive function by manipulating autophagy and triggering apoptosis [79]. In addition, cadmium is a toxic heavy metal that induces excessive mitochondrial fission and inhibits mitophagy, leading to apoptosis of mouse Leydig cells [19].

Oxidative stress can trigger autophagy in male germ cells, leading to two main scenarios. In the first scenario, autophagy helps degrade damaged proteins and organelles to maintain spermatogenic cell homeostasis, support spermatocyte meiosis and promote spermiogenesis, ultimately reducing the risk of testicular abnormalities. However, in the second scenario, excessive autophagy activation may result in increased degradation of proteins and organelles, leading to cellular dysfunction, decreased sperm count, abnormalities in spermatogenesis and reduced sperm motility [8].

## 5 Ferroptosis in Male Infertility

### 5.1 Ferroptosis Definition

Ferroptosis is a form of oxidative, iron‐dependent and non‐apoptotic cell death that is morphologically, biochemically and genetically distinct from other programmed cell death types such as apoptosis and autophagy [80]. Known inhibitors of apoptosis, autophagy, or necrosis do not repress ferroptosis. This type of cell death is characterised by enhanced mitochondrial membrane density and cell volume contraction [81]. Ferroptosis is induced by small molecules or conditions that lead to reduced biosynthesis of glutathione or the glutathione peroxidase 4 (GPX4) enzyme, resulting in iron‐dependent lipid ROS accumulation and depletion of PUFAs [12].

Indeed, ferroptosis affects metabolic function via the generation of both cytosolic and lipid ROS, independent of mitochondria but dependent on NADPH oxidases [82]. This process is induced by two groups of small molecules targeting two different key molecules. The first class includes erastin, sulfasalazine and sorafenib, which interfere with cystine and glutamate transport via system xc‐ [83]. System xc‐ is a membrane Na^+^‐dependent cysteine‐glutamate exchange antiporter consisting of a disulfide‐linked heterodimer with a light chain subunit (SLC7A11) and a heavy chain subunit (SLC3A2). This system transports glutamate to the extracellular space and cystine to intracellular space to form cysteine, an essential precursor for glutathione synthesis [84]. The second class of ferroptosis inducers is ferroptosis‐inducing agents (FINs), including RSL3, DPI7, DPI10 and DPI12, which inhibit GPX4 and give rise to the accumulation of lipid peroxides without reducing cellular glutathione [85]. GPX4 plays a crucial role in decreasing toxic lipid hydroperoxides (LOOH) to benign lipid alcohol (LOH) and acts as a critical regulator to prevent ferroptosis [86]. Studies have shown that systemic deletion of Gpx4 in mice results in embryonic lethality. Several research teams have attempted to knock down this gene, and the analysis of the cell death mechanism demonstrated lipid peroxidation in all knock‐down models [87]. Free cellular iron is believed to be the major mediator of lipid peroxide generation during ferroptosis. One possible explanation for the involvement of GPX4 in ferroptosis is that when GPX4 is inactivated, L‐OOHs accumulate and interact with iron, leading to the iron‐catalysed formation of lipid radicals (L‐O) that are lethal to the cell [86]. In addition to SLC7A11 and GPX4, several genes are involved in the ferroptotic cell death pathway, including mitochondrial genes such as VDAC1 and VDAC2. The expression of these genes is necessary but not sufficient for erastin‐induced cell death [80].

### 5.2 Role of Iron in Ferroptosis

In biological processes, controlling the free iron in cells is crucial. Under normal physiological conditions, iron circulates as Fe^3+^ bound to transferrin. Fe^3+^ enters cells through the transferrin receptor 1 (TFR1) on the cell membrane. Inside the cell, it is reduced to Fe^2+^ by the STEAP3 enzyme in the endosome. The Fe^2+^ is then released into the cytoplasmic iron pool by the divalent metal transporter 1 (DMT1) enzyme. Excess iron is stored in the form of ferritin, a complex composed of the ferritin light chain (FTL) and ferritin heavy chain 1 (FTH1). The Fe^2+^ can be exported back to the extracellular space through the membrane protein ferroportin. TRF1 and DMT1 can potentially induce ferroptosis by increasing free Fe^2+^ levels in the cytoplasm. On the other hand, the ferritin complex and ferroportin can prevent ferroptosis by decreasing free Fe^2+^ levels in the cytoplasm [88]. Yang et al. reported that ferroptosis‐sensitive cells exhibit up‐regulated TRF1 expression and down‐regulated FTH1 and FTL expression compared to ferroptosis‐resistant cells [89]. The exact mechanism by which iron triggers ferroptotic cell death is not fully understood, but iron chelators may shed some light on this process. Iron chelators can inhibit ferroptosis by blocking free iron from catalysing the formation of lipid or soluble radicals, which are involved in the oxidation of PUFAs. The Fenton reaction, facilitated by iron as a catalyst, may play a role in generating the highly toxic hydroxyl radical [81]. The Fenton reaction, leading to the formation of the hydroxyl radical, a highly toxic radical mediated by iron as a catalyst, might account for the role of iron in ferroptosis [88]. Additionally, iron chelators may directly deactivate iron‐dependent enzymes, such as the lipoxygenase family, which contribute to membrane lipid oxidation [81].

### 5.3 Role of ROS and Lipid Peroxidation in Ferroptosis

**FIGURE 4:**
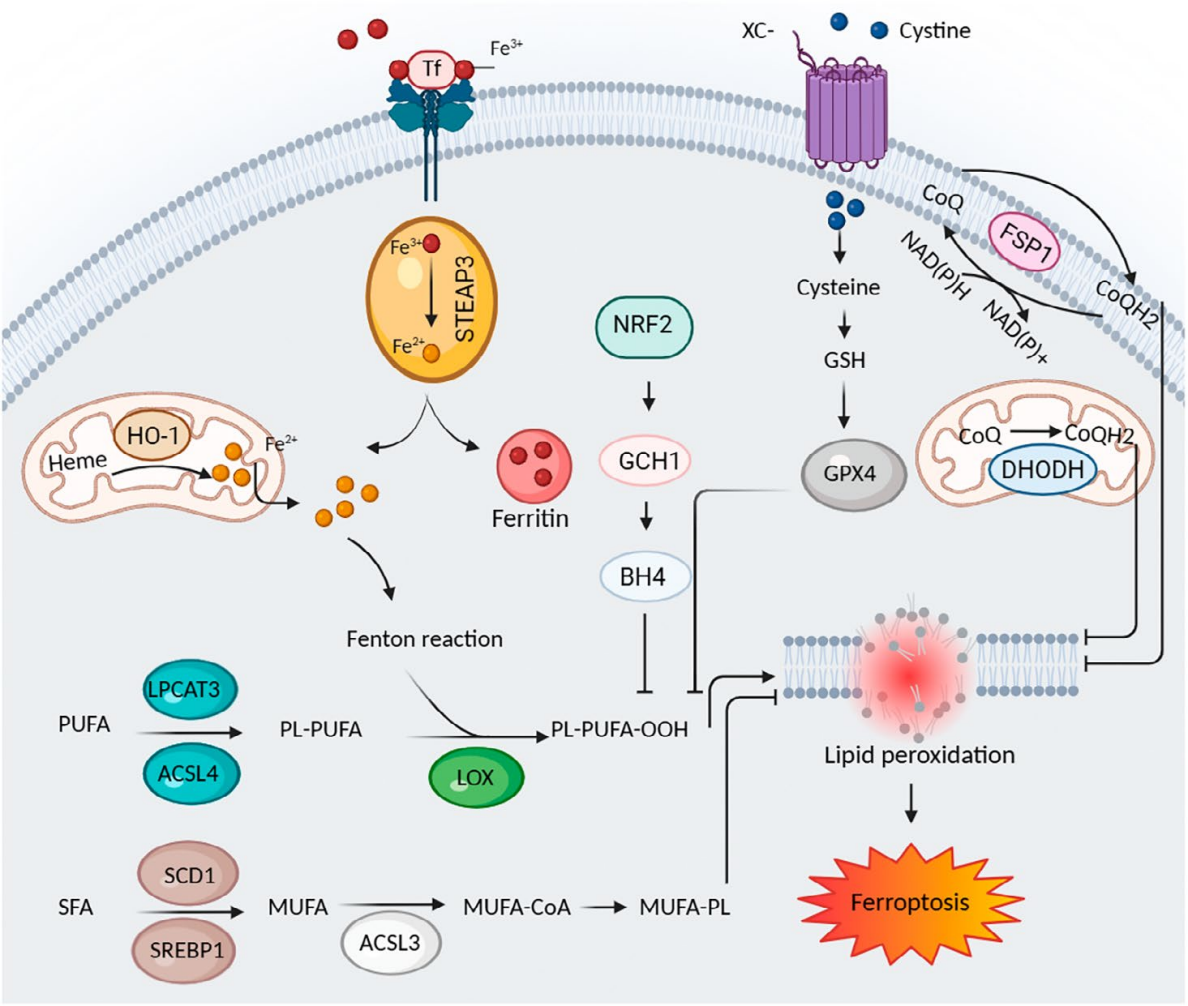
A schematic view of the ferroptosis pathway, illustrating the roles of iron, lipid peroxidation and ROS metabolism. System Xc‐ plays a crucial role in facilitating the uptake of cystine by cells. This process involves the conversion of cystine to cysteine by cystine reductase, which is an essential precursor in the synthesis of GSH. A reduction in GSH levels indirectly deactivates GPX4, leading to an accumulation of lipid peroxides that disrupt membrane integrity and ultimately result in ferroptosis. Transferrin‐bound circulating iron (Fe^3+^) is transported into cells via TFR1 and localises in endosomes, where it is converted to Fe^2+^. Simultaneously, HO‐1 increases intracellular iron levels by breaking down heme into Fe^2+^. The released Fe^2+^ enters a labile iron pool, with excess iron being stored in ferritin. The labile iron pool triggers the formation of •OH through the Fenton reaction, promoting ferroptosis by peroxidising phospholipid polyunsaturated fatty acids (PL‐PUFAs). The production of PL‐PUFAs by ACSL4 and LPCAT3 is crucial in promoting lipid peroxidation, which can be further oxidised to LOOH through either non‐enzymatic (Fenton reaction) or enzymatic (LOX) processes. SCD1 and SREBP1 convert SFAs into MUFAs, which are then transformed into acyl‐CoA esters by ACSL3. These esters can substitute PUFAs in cell membranes, thereby decreasing lipid peroxidation. FSP1 and DHODH reduce CoQ to its active form CoQH_2_ in the plasma and mitochondrial membranes, respectively, both contributing to the suppression of lipid peroxidation. Additionally, Nrf2 activates the GCH1/BH4 pathway to lower ROS production.

The origin of ROS in triggering ferroptosis is derived from multiple sources. Iron‐dependent Fenton reaction, NADPH‐mediated lipid peroxidation and GSH depletion are three pathways of ROS production. Additionally, glucose metabolism through mitochondrial fatty acids and glutamine to α‐ketoglutarate conversion also provides lipid precursors necessary for ROS generation in ferroptosis [88]. Lipid peroxidation is essential for inducing ferroptotic cell death. In this process, only one class of phospholipids, phosphatidylethanolamines (PE), is oxidised. Specifically, two PUFAs, arachidonoyl (AA) and adrenoyl (AdA)‐containing species of PEs are preferentially oxidised in the ferroptosis process [90]. To generate PUFAs‐containing PEs, PUFAs are acylated via acyl‐CoA synthetase long‐chain family member 4 (ACSL4) into PUFA‐CoA. Then, acylated PUFAs are esterified by a lysophosphatidylcholine acyl transferase (LPCAT3)‐dependent mechanism giving rise to PUFA‐PE (PL‐PUFA (PE)) [91]. Ferroptosis involves the oxidation of membrane PUFAs. Oxidation of PUFAs occurs through two processes including enzymatic oxidation and autoxidation, a free radical chain reaction. The former is catalysed by two groups of enzymes, the lipoxygenases (LOX) and cyclooxygenases families; however, only the lipoxygenase family has been implicated in ferroptotic cell death [87, 92]. Lipoxygenases such as arachidonate 15‐lipoxygenase (ALOX15) trigger lipid peroxidation by abstracting one labile hydrogen atom from a bis‐allylic position of a PUFA to generate a pentadienyl radical. Then, when a molecule of oxygen is added to this intermediate radical, it results in the formation of a peroxyl radical which is reduced by the lipoxygenases, giving rise to hydroperoxide products (PL‐PUFA‐OOH) [93]. These hydroperoxides are the main substrates of GPX4. The GPX4 counteracts lipid peroxidation as the first‐line defence and preferentially reduces toxic lipid hydroperoxides to their corresponding non‐toxic lipid alcohols while oxidising GSH to GSSG (Figure 4) [94]. Unlike PUFAs, monounsaturated fatty acids (MUFAs) contain fewer double bonds, making them less susceptible to lipid peroxidation and capable of protecting cells from ferroptosis by substituting PUFAs in membrane lipids. The strategy of some cells to resist ferroptosis is to increase the expression of the stearoyl‐CoA desaturase 1 (SCD1), an endoplasmic reticulum membrane‐bound protein, which synthesises MUFAs from saturated fatty acid (SFA), allowing them to raise MUFA levels and resist ferroptosis [95]. The expression of SCD1 is controlled by the transcription factor SREBP1, which also promotes genes like FASN involved in new lipid synthesis. Activation of the mTORC1 pathway or inhibition of AMPK can enhance SREBP1/SCD1‐dependent MUFA production, leading to increased resistance to ferroptosis. Acyl‐CoA synthetase long chain family member 3 (ACSL3) transforms MUFAs produced by SCD1 into acyl‐CoA esters, which are then integrated into membrane phospholipids. This process helps cells resist ferroptosis by stabilising their membranes. This adaptation reflects metabolic reprogramming, emphasising the need to study the PUFA–MUFA balance to better understand lipid metabolism's role in ferroptosis [96]. Recent discoveries have revealed a network consisting of ferroptosis‐suppressor‐protein 1 (FSP1), ubiquinone (CoQ10) and nitrate reductase (NAD(P)H) as an alternative protection against lipid peroxidation even in the absence of GPX4. FSP1 was identified as an anti‐ferroptotic gene that can compensate for GPX4 loss. This FSP1 action is mediated via CoQ10 and NAD(P)H [97, 98]. In addition to the plasma membrane‐associated oxidoreductase FSP1, recent studies have identified a mitochondria‐specific ferroptosis defence pathway mediated by the dihydroorotate dehydrogenase (DHODH)–ubiquinol (CoQH2) system. DHODH, an essential enzyme in de novo pyrimidine biosynthesis, cooperates with GPX4 to suppress ferroptosis in the mitochondrial inner membrane by attenuating lipid peroxidation [99]. Recent studies recognise GCH1/BH4 as a separate ferroptosis regulatory system. Under oxidative stress, overexpression of Nrf2 activates the GCH1/BH4 pathway, increasing GCH1 expression. This upregulation restores intracellular BH4 levels, reducing ROS production and alleviating oxidative stress as part of an antioxidant defence mechanism [100]. Emerging evidence also shows that overexpression of heme oxygenase 1 (HO‐1) raises intracellular iron levels, through cleavage of heme to Fe2+, resulting in redox balance disruption and ferroptosis induction, while Nrf2 helps reduce oxidative stress and ferroptosis by downregulating HO‐1 [101]. Non‐enzymatic Fenton reaction or autoxidation process is induced by any reactive species that can abstract a hydrogen atom from the bis‐allylic position of a PUFA, such as hydroperoxyl, hydroxyl and alkoxyl radicals to generate a pentadienyl radical. This intermediate radical reacts with molecular oxygen, giving rise to a chain‐carrying peroxyl radical that can propagate a chain reaction by abstracting a hydrogen atom from an adjacent lipid. This autocatalytic process of radicals can initiate a new radical chain [87]. The mechanism by which the oxidation of plasma membrane PUFAs leads to ferroptotic cell death remains unclear; however, PUFAs oxidation may result in the formation of gaps in the plasma membrane, leading to the loss of ionic homeostasis. Besides, the accumulation of oxidised PUFAs or derivative fragments could directly inactivate vital intracellular proteins [91].

### 5.4 Role of Amino Acid Metabolism in Ferroptosis

Among amino acids, glutamate, glycine and cysteine are key players in ferroptosis regulation because they combine to form GSH, a major ferroptosis inhibitor. Excess extracellular glutamate blocks system Xc−, reducing cystine uptake and disrupting the antioxidant balance, which triggers ferroptosis. Cysteine is crucial for GSH synthesis and GPX4 activity; its shortage decreases GSH levels, enhances lipid peroxidation and induces ferroptosis [102]. Interestingly, DeNicola et al. showed that the enzyme GCLC can protect cells from cysteine‐deprivation‐induced ferroptosis by generating γ‐glutamyl peptides, a process strengthened by NRF2 activation [103]. Moreover, glycine and cysteine are derived from serine metabolism. When serine levels drop, GSH synthesis decreases, leading to reduced GPX4 stability and ultimately triggering ferroptosis [104]. The essential amino acid methionine is converted into S‐adenosylmethionine (SAM), which donates methyl groups for cysteine synthesis. This process helps maintain GPX4 activity and consequently prevents ferroptosis [105].

## 6 Interaction Between Autophagy, Apoptosis and Ferroptosis

**FIGURE 5:**
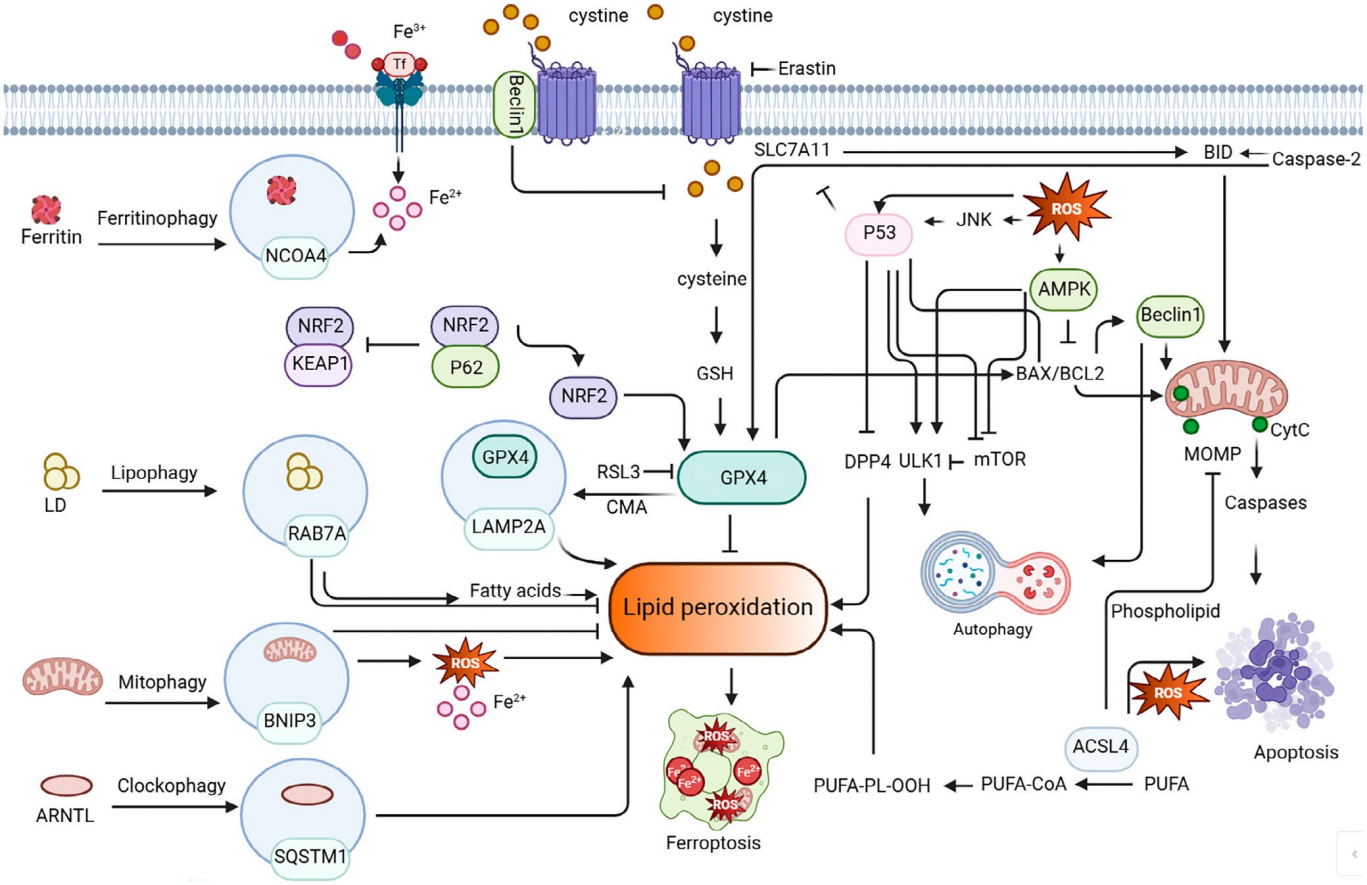
Intersecting pathways of apoptosis, autophagy, ferroptosis and oxidative stress. This figure illustrates the intricate crosstalk between apoptosis, autophagy, ferroptosis and oxidative stress, with a focus on key molecular players and regulatory mechanisms. Apoptosis–Ferroptosis Interaction: p53 serves as a central regulator, promoting ferroptosis by inhibiting SLC7A11 and GSH synthesis, while also suppressing ferroptosis through interaction with DPP4. ACSL4 links lipid metabolism to both pathways by modulating fatty acyl‐CoA levels and oxidative stress. Inhibitors of GPX4, such as RSL3, can induce overlapping apoptotic and ferroptotic features, including cytochrome c release and caspase activation, while BID and caspase‐2 connect mitochondrial apoptosis to ferroptotic regulation. Autophagy–Ferroptosis Interaction: Autophagy enhances ferroptosis through selective processes such as ferritinophagy (iron release via NCOA4), lipophagy (degradation of lipid droplets), clockophagy (ARNTL degradation), mitophagy (ROS regulation via BNIP3/NIX) and CMA that degrades GPX4. Beclin‐1 and the p62–KEAP1–NRF2 axis act as key regulators, linking autophagy machinery to redox balance and ferroptosis sensitivity. Autophagy–Apoptosis Interaction: Beclin‐1 functions as a key mediator between autophagy and apoptosis, being cleaved by caspases and inhibited by Bcl‐2/BCL‐XL. AMPK promotes apoptosis by suppressing Bcl‐2, while p53 regulates both apoptosis (via pro‐apoptotic genes) and autophagy (through PTEN–AKT–mTORC1 signalling).

Complex interactions among apoptosis, autophagy, ferroptosis and oxidative stress are depicted in Figure 5, highlighting the major molecular components and regulatory pathways involved.

### 6.1 Interaction Between Apoptosis and Ferroptosis

Ferroptosis and apoptosis, while typically distinct, can intersect and influence each other. An important intersection between apoptosis and ferroptosis is mediated by p53. While p53's conventional roles in cell cycle arrest, senescence and apoptosis are well established as key stress response checkpoints, p53 also regulates the ferroptosis response through its metabolic targets. In the presence of ferroptosis inducers, such as GPX4 inhibitors or high levels of ROS, p53 modulates ferroptosis by directly suppressing the SLC7A11 gene, which limits GSH production and promotes ferroptosis [106]. p53 can suppress ferroptosis in a transcription‐independent manner by interacting with dipeptidyl‐peptidase‐4 (DPP‐4), a regulator of ferroptosis and lipid metabolism, highlighting its dual role in ferroptosis regulation. Another intersection between apoptosis and ferroptosis involves ACSL4. It promotes apoptosis by elevating fatty acyl‐CoA levels, increasing oxidative stress and activating intrinsic apoptosis, as well as by directly interacting with apoptotic signalling proteins. Conversely, fatty acid oxidation activates STAT3, which increases ACSL4 expression. This upregulation increases phospholipid synthesis and leads to enhancing mitochondrial membrane integrity, inhibiting mitochondrial outer membrane permeabilisation (MOMP) and suppressing apoptosis [54].

A recent study by Qiu and colleagues demonstrated that inhibition of GPX4 by RSL3 induces both ferroptotic and apoptotic features, including cell swelling, typical of ferroptosis, along with membrane blebbing, characteristic of apoptosis. RSL3 treatment leads to cytochrome‐c release, caspase‐3 activation and PARP cleavage, confirming apoptosis, which is BAX/BAK‐dependent. Additionally, targeting BCL‐2 or BCL‐XL with BH3‐mimetics alongside GPX4 inhibition often enhances cell death and shifts the outcome from ferroptosis to apoptosis [107]. Moreover, erastin‐induced ferroptosis is linked to mitochondrial damage, including BID transactivation, loss of membrane potential, mitochondrial fragmentation and reduced ATP levels. So that, inhibition of BID preserved mitochondrial integrity and protected against ferroptosis, suggesting that mitochondrial transactivation of BID is a critical step connecting ferroptosis to mitochondrial damage in oxidative cell death [84]. Caspase‐2 which cleaves and activates Bid and induces MOMP in apoptosis pathway, acts as a negative regulator of ferroptosis by stabilising GPX4. Under oxidative stress, the absence of caspase‐2 destabilises GPX4, making cells more susceptible to ferroptotic cell death [108].

### 6.2 Interaction Between Autophagy and Ferroptosis

Autophagy activity increases in different cell types when exposed to classical ferroptosis inducers like erastin and RSL3. Although moderate autophagy likely developed as a survival mechanism, excessive autophagy—particularly selective forms including ferritinophagy, lipophagy, clockophagy, mitophagy and chaperone‐mediated autophagy (CMA)—and disrupted lysosomal function can contribute to ferroptotic cell death. The principal categories of selective autophagy, along with key regulators of the autophagic machinery, are delineated herein as pivotal contributors to the induction of ferroptosis [109]. Nuclear Receptor Cofactor 4 (NCOA4) is a specific receptor for ferritin that allows ferritinophagy (ferritin phagocytosis). When NCOA4 is silenced, ferritin levels rise and labile iron levels fall. When there is enough iron in the body, HERC2 (a large E3 ubiquitin‐protein ligase) ubiquitinates NCOA4 and sends it to the proteasome to be broken down. This helps iron be stored in ferritin. In contrast, in iron‐deficient environments, NCOA4 adopts its apo protein configuration and interacts with ferritin, promoting ferritinophagy and the release of stored iron. Meanwhile, the increase of free iron levels provides the possibility of ferroptosis [110].

Lipophagy—the autophagic process that degraded lipid droplets (LDs) within cells—facilitates ferroptotic cell death triggered by RSL3 in hepatocyte cells. The LDs initially accumulate during the early phase of ferroptosis but decline in the later stages. Starvation also triggers lipophagy, causing LDs to bind with autophagosomes and release free fatty acids, which are key for lipid peroxidation in ferroptosis [111]. Notably, increasing lipid storage through TPD52 or inhibiting lipid degradation via ATG5 and RAB7A (a cargo receptor for lipid droplet autophagy) can block RSL3‐induced lipid peroxidation and the resulting ferroptosis, both in vitro and in vivo [112].

The other form of selective autophagy is SQSTM1‐dependent clockophagy that targets the circadian regulator ARNTL (BMAL1) for degradation. The loss of ARNTL disrupts cellular redox homeostasis, leading to increased lipid peroxidation and heightened sensitivity to ferroptosis. This process links the circadian clock machinery to the regulation of ferroptotic cell death through an SQSTM1‐mediated pathway [113]. Mitophagy, the selective degradation of damaged mitochondria, preserves mitochondrial function. The role of mitophagy in ferroptosis induction is complex. Mitophagy, mediated by receptors such as BNIP3 and NIX, maintains mitochondrial homeostasis and limits the accumulation of mitochondrial ROS, a key driver of lipid peroxidation and ferroptosis [114]. In the early stages of iron overload, mitophagy can sequester excess iron into mitophagosomes, reducing ROS generation and limiting ferroptosis. However, severe mitochondrial damage from excessive iron triggers additional mitophagy, releasing more iron and promoting lipid peroxidation, thereby enhancing ferroptotic cell death [115].

Another form of autophagy that regulates ferroptosis is CMA. Among the key antioxidants in ferroptosis, GPX4 plays a central role. In this process, molecular chaperones identify specific amino acid sequences within target proteins, bind them to lysosome‐associated membrane protein type 2A (LAMP2A) and transport them into the lysosome for degradation. During ferroptosis, activation leads to elevated LAMP2A expression and induces CMA in an HSP90‐dependent manner, which promotes the breakdown of GPX4 and thereby enhances ferroptosis [111].

Apart from the specific types of autophagy, there are various proteins that act as crucial regulators of autophagy in ferroptosis. Beclin1 (a homologue of yeast ATG6), as a crucial regulator in the initiation of autophagy, can bind to SLC7A11/system Xc and suppress the transport activity of tumour cells to a type 1 ferroptosis inducer. When phosphorylated by AMPK, Beclin1 limits the SLC7A11/system Xc complex and inhibits ferroptosis in cells [116].

NRF2 as a key antioxidant defence factor, regulated by KEAP1 under normal conditions, prevents its activation. In oxidative stress, NRF2 is released from KEAP1, translocates to the nucleus and activates antioxidant genes. P62, an autophagy receptor, competes with KEAP1 to stabilise NRF2, enabling its role in regulating ferroptosis through the p62‐KEAP1‐NRF2 pathway (101).

### 6.3 Interaction Between Autophagy and Apoptosis

Autophagy and apoptosis can either work together or oppose each other, depending on the influencing factors and surrounding conditions. Beclin1, a key regulator of autophagy, interacts with both autophagy and apoptotic proteins, playing a vital role in regulating the crosstalk between autophagy and apoptosis. Beclin‐1 is cleaved by apoptotic caspases and anti‐apoptotic proteins Bcl‐2 or BCL‐xL, leading to apoptosis and suppression of autophagy [117]. Additionally, AMPK promotes apoptosis by downregulating the expression of anti‐apoptotic Bcl‐2 proteins. p53 promotes apoptosis by activating pro‐apoptotic and inhibiting anti‐apoptotic proteins. It also promotes autophagy by inhibiting the AKT pathway via PTEN. Since mTORC1, which lies downstream of PI3K/AKT, blocks autophagy by inhibiting factors like ULK1, p53 indirectly enhances autophagy by suppressing mTORC1 activity [118].

## 7 Ferroptotic Cell Death in Male Infertility

The reciprocal relationship between diminished GPX4 activity and heightened lipid peroxidation as well as iron overload establishes a self‐perpetuating cycle that exacerbates cellular injury within the testis. Although direct experimental validation remains limited, converging indirect evidence—encompassing oxidative stress, reactive oxygen species (ROS) accumulation, iron dyshomeostasis and disrupted glutathione metabolism—strongly implicates ferroptosis as a contributing factor in testicular dysfunction and impaired sperm quality. Given the high content of polyunsaturated fatty acids in spermatozoa, these cells exhibit exceptional vulnerability to oxidative insult and ferroptotic damage [119]. Recent studies have demonstrated that iron overload and mitochondrial dysfunction induce elevated oxidative stress leading to lipid peroxidation and ferroptosis in pachytene spermatocytes, round spermatids and ultimately impair male reproductive function. Sertoli cells are also susceptible to ferroptotic cell death. Moreover, disruptions in the antioxidant defence system, together with lifestyle factors, further contribute to the induction of ferroptosis [120, 121]. In recent years, numerous clinical and preclinical studies have investigated ferroptosis in testicular cells, some of which are summarised below.

### 7.1 Preclinical Data Related to Ferroptosis in Male Reproduction

For the first time, Bromfield and her colleague explored the role of ferroptotic cell death in mouse germ cells following exposure of round spermatids and pachytene spermatocytes to acute oxidative stress conditions such as 4HNE, erastin and RSL3. They found that the protein levels of key ferroptotic regulators, ALOX15 and ACSL4 significantly increased, while GPX4 protein levels decreased compared to the untreated control. They demonstrated that this induced ferroptotic pathway could be attenuated by deferoxamine and ferrostatin‐1 as ferroptosis inhibitors as well as by PD146176 and Rosiglitazone as inhibitors of ALOX15 and ACSL4 respectively. Therefore, their research supports the important role of ALOX15 and ACSL4 in ferroptosis of mouse round spermatids under acute oxidative stress conditions [10].

In another study conducted by Han et al., ferroptosis was identified as a crucial process responsible for the decline in sperm count and motility in Nrf2 knocked‐out male mice (Nrf2−/−). The decline in sperm concentration and motility was observed in these mice. Furthermore, the symptoms of oligospermia in Nrf2−/− mice were alleviated by the administration of the ferroptosis inhibitor, ferrostatin‐1. These findings suggest that targeting ferroptosis could be a promising approach to address oligospermia in males with reduced Nrf2 expression in their sperm [122]. In addition to germ cells, ferroptosis has been demonstrated to be a critical and dynamic form of cell death induced by testicular ischemia–reperfusion injury in Sertoli cells. Ferroptotic cell death triggered by this injury is associated with elevated lipid ROS, iron accumulation, GSH depletion, GPX4 inactivation and downregulation of ferroportin. This process can be blocked by N‐acetylcysteine (a ROS inhibitor), Liproxstatin‐1 (a lipid peroxidation inhibitor) and the iron chelator deferoxamine [123].

### 7.2 Clinical Data Related to Ferroptosis in Male Infertility

Induction of ferroptosis by AA in human spermatozoa led to elevated cytosolic and mitochondrial ROS production, along with impaired mitochondrial membrane potential (ΔΨm), reduced viability and decreased motility. These changes were linked to biochemical and molecular markers of ferroptotic cell death, including increased iron content in the form of ferrous (Fe^2+^) ions, upregulation of SLC7A11, ACSL4 and IREB2, and a downregulation of GPX4. Additionally, lipid peroxidation levels were significantly higher in the AA‐treated group compared to the untreated control [124].

There are several distinct hallmarks of ferroptosis in varicocele‐related male infertility, including decreased levels of antioxidant components such as GSH [125], high levels of lipid peroxidation, and related byproducts like MDA and 4HNE [126], as well as excessive iron toxicity [127]. While iron is essential for testosterone synthesis and spermatogenesis, an excess of iron can negatively impact sperm quality by inducing oxidative stress and lipid peroxidation, leading to male reproductive dysfunction [128, 129]. Sun et al. investigated the impact of bilateral varicocele on semen quality and the molecular mechanisms involving ferroptosis in infertile men. Their findings revealed that patients with bilateral varicocele exhibited significantly lower sperm progressive motility and motility rate. Elevated levels of ROS and iron were identified as key factors leading to ferroptosis in these patients [125].

In a recent study, key molecular events of ferroptosis were examined in infertile men with urogenital infections and varicocele, compared with fertile controls. Seminal transferrin and ferritin showed positive correlations with F2‐isoprostanes (F2‐IsoPs), an oxidative stress marker that negatively correlated with sperm parameters, suggesting that iron metabolism dysregulation may impair semen quality by promoting lipid peroxidation. Moreover, ACSL4 levels were significantly elevated in the infertile group, and sperm ACSL4 concentration positively correlated with F2‐IsoPs [130]. In a study by our team, the induction of varicocele in Wistar rats resulted in elevated lipid peroxidation and iron accumulation. However, the relative expression of ferroptotic molecular markers (Nrf2, SLC7A11, P53 and p‐Jnk) did not exhibit significant changes. This discrepancy may be attributed to the mosaic nature of the varicocele testis, which could mask the detection of ferroptosis molecular markers [131]. Oxidative stress is a key factor in blood–testis barrier (BTB) disruption during cryptorchidism. Zeng et al. showed that blocking the Nrf2/Keap1/HO‐1 pathway reduced GPX4 and FPN1 while increasing FTL expression. Their results confirm that inhibiting ferroptosis alleviates OS‐induced BTB damage by restoring tight junction proteins [132]. The level of ferroptosis was further examined in asthenozoospermia patients. Compared with the normozoospermic group, asthenozoospermic individuals exhibited significantly higher ROS, MDA and iron levels, accompanied by reduced mitochondrial membrane potential. Moreover, the downregulation of GPX4 and SLC7A11 expression suggested an increased susceptibility to ferroptosis [132]. Integrative bioinformatics analysis revealed differential expression of ferroptosis‐related genes between the non‐obstructive azoospermia (iNOA) group and controls, suggesting their potential as biomarkers for the diagnosis and treatment of azoospermia [133, 134].

## 8 Induction of Testicular Ferroptosis by Drugs, Lifestyle and Environmental Factors

Unhealthy lifestyle choices, frequent use of certain medications and environmental exposure to heavy metals resulting from human activities can lead to testicular damage and contribute to male infertility. In a discovery, Ou and his colleagues revealed an association between cigarette smoking and high levels of ferroptosis in the seminal plasma of infertile heavy smokers and GC‐2Spd cells exposed to cigarette smoke condensate. According to this study, the GSH level was significantly decreased, while the levels of iron and lipid ROS were increased dramatically in the seminal plasma of the heavy smoker group relative to non‐smokers as well as in GC‐2Spd cells after exposure to cigarette smoke condensate. Treatment of GC‐2Spd cells with Ferrostatin‐1 resulted in a meaningful elevation in GSH levels and GPX4 protein expression and a significant reduction in lipid ROS and iron levels [135]. Ferroptosis is also proposed as a major cause of germ cell death in busulfan‐induced oligospermia in mice. Busulfan is an anti‐cancer chemotherapeutic drug that has adverse effects on spermatogenesis and may lead to male infertility. Busulfan triggers ferroptosis in mouse germ cells by decreasing the expression of GPX4, nuclear factor‐E2‐related factor 2 (Nrf2) and ferroportin‐1. Suppression of ferroptosis using ferrostatin‐1 or deferoxamine in busulfan‐treated mice could reverse busulfan‐induced oligospermia by upregulating GPX4, Nrf2 and ferroportin‐1 [136]. Moreover, ferroptosis, rather than other forms of programmed cell death, plays a key role in cisplatin‐induced testicular damage and Sertoli cell loss. Cisplatin induces ferroptosis through N6‐methyladenosine (m6A), one of the most abundant RNA modifications, which alters the expression of ferroptosis‐related genes in a cisplatin exposure mouse model. In Sertoli (TM4) cells, cisplatin‐induced ferroptosis is independent of GPX4 but is regulated by SLC7A11 and ALOX12, both of which are modulated in an m6A‐dependent manner by the methyltransferase METTL3 [137]. Furthermore, anti‐PD‐1 therapy has been shown to compromise male reproductive function, manifesting as reduced sperm concentration, altered gonadal hormone levels and disruption of BTB integrity. These effects are mediated, at least in part, by ferroptosis, while suppression of the NRF2 antioxidant pathway further contributes to the observed testicular dysfunction [138]. Cadmium (Cd) exposure at 5 ppm resulted in substantial germ cell loss, reduced meiotic index and decreased testicular weight. This concentration of Cd also promoted iron accumulation, elevated oxidative stress markers such as MDA and 3‐nitrotyrosine (3‐NT) and suppressed the expression of antioxidant regulators including Nrf2, HO‐1 and SOD2. Moreover, Cd exposure significantly downregulated SLC7A11, a ferroptosis marker, suggesting that Cd‐induced testicular ferroptosis is likely mediated by impaired iron export [139]. Di‐(2‐ethylhexyl) phthalate (DEHP), a widely utilised plasticiser in daily human life, has been reported to disturb testosterone regulation and compromise BTB integrity. This disruption promotes ferroptosis in Sertoli cells, mediated through the p38α–lipid ROS signalling cascade [140]. Moreover, DEHP triggered blood‐testis barrier (BTB) dysfunction by targeting the transferrin receptor (TfRC), as TfRC knockdown blocked MEHP‐induced ferroptosis by reducing mitochondrial and intracellular Fe^2^+ levels [141]. Fine particulate matter (PM2.5), a significant air pollutant, also induces ferroptosis in spermatocytes through iron overload and lipid peroxidation. This ferroptosis can be reversed with treatment using the iron chelator deferoxamine mesylate and the lipid peroxidation inhibitor ferrostatin‐1. The lipid metabolic genes Acsl4 and Aloxe3, as well as the antioxidant gene Gpx4 are key target genes in this event [142]. Acrolein, a widespread environmental pollutant, induces ferroptotic death in Sertoli cells by depleting GSH, downregulating SLC7A11, elevating intracellular Fe^2+^ and promoting lipid peroxidation [143]. In addition, exposure to arsenite (which is commonly found in the environment) in the mouse testis promotes iron accumulation and mitochondrial impairment, concomitantly activating ferroptosis‐associated signalling pathways. Inhibition of ferroptosis by ferrostatin‐1 significantly mitigates arsenite‐induced testicular cell death [144]. Tripterygium wilfordii polyglycoside (TWP), a traditional Chinese medicine widely prescribed for rheumatoid arthritis, is clinically limited by its pronounced reproductive toxicity. Qin et al., through integrated analyses of differentially expressed genes and altered metabolites, demonstrated that disruptions in glutathione metabolism and the activation of ferroptosis are key mechanisms underlying TWP‐induced testicular injury [145]. Amorphous silica nanoparticles (SiNPs), widely used in cosmetics, food additives, pharmaceuticals and drug delivery systems, have been increasingly recognised as a risk to male reproductive health. Exposure to SiNPs led to significant activation of oxidative stress, ferroptosis and cell cycle pathways. In GC‐2spd cells, SiNPs caused iron accumulation and markedly reduced the levels of GPX4 and GSH, while increasing MDA and HO‐1 expression. Furthermore, BRCA1 was identified as a regulator of GPX4, playing a key role in SiNP‐induced ferroptosis [146].

### 8.1 The Relationship Among Apoptosis, Autophagy and Ferroptosis in Male Reproductive Diseases

All three forms of programmed cell death triggered by ROS are closely regulated and have adapted to diverse environments. A limited number of studies have explored the interaction between these programmed cell death pathways in response to species‐specific conditions or toxins in testicular cells. Despite the lack of evidence for a direct relationship between apoptosis and autophagy with ferroptosis, their concurrent occurrence has been observed in toxicity with some toxins in the testis [19, 78, 147]. A recent study by Guo et al. revealed the dual role of the toxin copper sulfate in testicular injury and spermatogenesis disruption. Autophagy protects the testis from copper sulfate‐induced oxidative damage and apoptosis. Autophagy inhibition in mouse‐derived spermatogonia cells reduces cellular survival and amplifies ROS production and apoptosis. However, autophagy exacerbates copper sulfate toxicity by promoting ferroptosis [88]. Furthermore, Diphenyl phosphate (DPhP), a widely used plasticiser, inhibits autophagy and promotes apoptosis in GC‐2spd(ts) cells by reducing Nrf2/p53 signalling [148]. Another study revealed that HT‐2 toxin, a mycotoxin in food and water, impairs Leydig cell proliferation and testosterone secretion by inducing ferroptosis and apoptosis through ROS‐mediated lipid peroxidation [147].

The interaction between autophagy and ferroptosis in testicular cells has been investigated in a few studies. Li and colleagues demonstrated that excessive zinc exposure triggers ferroptotic cell death by regulating mitophagy, leading to porcine testicular dysfunction. The zinc‐induced ferroptosis is characterised by a decrease in the expression of SLC7A11, GPX4 and ferritin. These effects were reversed by treating the cells with ferrostatin‐1 or ML‐210. Besides, this treatment significantly upregulated the expression of mitophagy‐related proteins, including PINK, Parkin, ATG5 and LC3‐II/LC3‐I. These findings suggest that mitophagy may be involved in zinc‐induced ferroptosis [149]. Recently, a ferroptosis model in mouse Sertoli cells, induced by erastin, showed that ferroptosis activation caused cytoskeletal disruption and BTB damage, along with excessive autophagy. Inhibition of autophagy restored BTB integrity, highlighting the interplay between ferroptosis and autophagy. Transcriptome analysis identified RAB3IL1 as a key ferroptosis regulator, with its knockdown promoting ferroptosis and BTB disruption. These findings suggest that the ferroptosis‐autophagy axis, regulated by RAB3IL1, is crucial for maintaining the spermatogenic microenvironment and could be a potential therapeutic target in male infertility [149]. Busulfan not only triggers ferroptosis but also promotes ferritinophagy. Inhibition of autophagy mitigates busulfan‐induced FTH1 degradation, thereby preventing ferroptosis in GC‐1 spg cells and testicular spermatogonia. This intervention ultimately reduces busulfan‐induced testicular damage and spermatogenesis disruptions [150]. The relationship between autophagy and ferroptosis is complex and multifaceted, with ongoing research aimed at unravelling its complexity. While ferroptosis shares some similarities with autophagy, further studies are needed to fully understand the nature of their relationship [151].

## 9 The Impact of Antioxidant Treatment on Oxidative Stress–Induced Programmed Cell Death in Male Infertility

Programmed cell death related to ROS involves the oxidation of specific types of lipids, suggesting antioxidants may reverse certain male reproductive disorders. Loss of GPx4 triggers lipid peroxidation–driven cell death across multiple mouse tissues, including the testis. Notably, vitamin E supplementation in tissue‐specific GPx4 knockout mice alleviated damage in this organ, underscoring the protective role of antioxidant defences [152].

Ferroptosis has been implicated in diabetes‐induced testicular dysfunction and is considered a central mediator linking metabolic dysregulation to reproductive damage in diabetes. Two separate studies have demonstrated that semaglutide, a GLP‐1 receptor agonist widely used for type 2 diabetes, and Guhan Yangsheng Jing (GHYSJ), a traditional Chinese patent medicine, exert protective effects on testicular function in diabetic mouse models. Treatment with these agents significantly alleviated testicular damage, as evidenced by reduced lipid peroxidation and improved regulation of ferroptosis‐related markers [153, 154]. Guilu‐Erxian‐Glue (GLEXG), a traditional Chinese formula, was evaluated for its protective effects in a rat model of oligoasthenospermia induced by TWP. High‐dose GLEXG significantly improved sperm quality, reduced oxidative stress and iron accumulation and restored mitochondrial function. These benefits were mediated through activation of the Keap1/Nrf2/GPX4 pathway, suggesting ferroptosis resistance as a novel therapeutic mechanism [155]. In a study conducted by our group, alpha‐lipoic acid (ALA), possessing both antioxidant and anti‐ferroptotic properties, was shown to reduce iron accumulation and elevate GSH levels in a rat model of varicocele [131]. Studies have demonstrated that deferoxamine significantly attenuates ferroptotic cell death in the testis. Moreover, subsequent investigations revealed that hydrogen sulfide (H_2_S), owing to its antioxidant and acrolein‐detoxifying properties, confers protection to Sertoli cells against acrolein‐induced ferroptosis [143]. Additionally, deferoxamine and ferrostatin‐1 reduced silica nanoparticle–induced lipid peroxidation, iron accumulation and cytotoxic effects in GC‐2spd cells [146]. Another compound that enhances antioxidant capacity is curcumin. Curcumin counteracted plasticiser‐induced apoptosis and alleviated reproductive toxicity by elevating Nrf2 levels and promoting autophagy through the Nrf2/p53 signalling pathway [148]. Moreover, treatment with curcumin, a specific activator of BRCA1, significantly mitigated the SiNP‐induced suppression of BRCA1 and GPX4 expression [146]. Quercetin is another natural antioxidant used to protect testicular cells. It mitigates Cd‐induced testicular damage by reducing GSH and MDA levels, inhibiting apoptosis and regulating the mitochondrial Cyt‐c/Caspase‐9/Caspase‐3/Bax/Bcl‐2 pathway [156]. N‐acetylcysteine alleviated histopathological alterations, enhanced antioxidant defences by increasing superoxide dismutase and glutathione peroxidase while reducing MDA, and modulated apoptosis‐related gene expression, thereby decreasing germ cell apoptosis in a rat model of varicocele‐induced testicular hypoxia [157]. *Lycium barbarum* polysaccharide mitigates DEHP‐induced testicular injury associated with ferroptosis by activating the NRF2/SLC7A11/GPX4 pathway, thereby restoring Sertoli cell function and alleviating male reproductive damage [158]. Resveratrol, a plant‐derived compound, mitigates anti‐PD‐1‐induced testicular toxicity by exerting antioxidant and anti‐ferroptotic effects, notably through NRF2 activation and the maintenance of iron and lipid homeostasis [138]. Studies show that the ferroptosis inhibitor Trolox preserves the quality of frozen‐stored ram spermatozoa by reducing lipid peroxidation and improving membrane and mitochondrial stability [159]. Astragalin, a major active compound in the traditional Chinese medicine Wuziyanzong Pill, is clinically used to treat male infertility. In a study by Cai and colle9agues, Astragalin was found to improve sperm quality and serum sex hormone levels in oligoasthenospermic rats. The mechanism of action involves promoting the nuclear translocation of Nrf2, reducing the expression of testicular Fe^2+^ and TFR1, upregulating testicular SLC7A11, GPX4 and FTH1, and inhibiting testicular ferroptosis [160] (Table 1).

**TABLE 1:** Summary of possible antioxidant treatments targeting oxidative stress–mediated programmed cell death in male infertility.

Drugs	Subject	Mechanism	References
Vitamin E	Mice	Suppresses phospholipid peroxidation	[152]
Semaglutide	Diabetic Mice	Reduces lipid peroxidation	[153]
Guhan Yangsheng Jing	Diabetic Rats	Reduces lipid peroxidation	[154]
Guilu‐Erxian‐Glue	Oligoasthenospermic Rats	Reduces oxidative stress and iron accumulation, through Keap1/Nrf2/GPX4 pathway	[155]
Alpha‐lipoic acid	Rat model of varicocele	Reduces iron accumulation and elevates GSH	[131]
Deferoxamine Ferrostatin‐1	GC‐2spd cells	Reduces silica nanoparticle–induced lipid peroxidation and iron accumulation	[146]
Hydrogen sulfide	TM4 cells	Protects to Sertoli cells against acrolein‐induced ferroptosis	[143]
Curcumin	GC‐2spd cells	Attenuates diphenyl phosphate‐induced apoptosis through activated autophagy via the Nrf2/P53 pathway	[148]
Curcumin	GC‐2spd cells	Mitigates the SiNP‐induced suppression of BRCA1 and GPX4 expression	[146]
Quercetin	Rats	Mitigates Cd‐induced testicular damage by reducing GSH and MDA levels, inhibiting apoptosis and regulating the mitochondrial Cyt‐c/Caspase‐9/Caspase‐3/Bax/Bcl‐2 pathway	[156]
N‐acetylcysteine	Rat model of varicocele	Increasing superoxide dismutase and glutathione peroxidase while reducing MDA	[157]
*Lycium barbarum*	Mice	Mitigates DEHP‐induced ferroptosis by activating the NRF2/SLC7A11/GPX4 pathway	[158]
Resveratrol	Mice	Mitigates anti‐PD‐1‐induced testicular ferroptosis, through NRF2 activation and the maintenance of iron and lipid homeostasis	[138]
Trolox	Frozen‐stored ram spermatozoa	Reduces lipid peroxidation and improves membrane and mitochondrial stability	[159]
Wuziyanzong	Oligoasthenospermic Rats	Promotes the nuclear translocation of Nrf2, reduces the expression of testicular Fe^2+^ and TFR1 and upregulates testicular SLC7A11, GPX4 and FTH1	[160]

## 10 Conclusion and Prospects for Future Research

Until recently, it was believed that apoptosis and autophagy were the only forms of programmed cell death in response to ROS accumulation in testicular cells that could lead to male reproductive dysfunction. However, recent evidence suggests that ferroptotic cell death is the third form of programmed cell death in this context. In addition to the reduction in antioxidant levels and the elevation of lipid peroxidation products, the accumulation of free iron levels through the Fenton reaction is another key process in ferroptosis that occurs during male reproductive dysfunction in testicular cells. The mechanisms of programmed cell death, including apoptosis, autophagy and ferroptosis, exhibit intricate interactions and complexities, with ongoing research striving to unravel their relationships. Although various chemical and herbal agents have been explored to mitigate oxidative stress, lipid peroxidation, iron overload and associated forms of cell death in the context of male infertility, current research remains at an early stage. The development and investigation of novel therapeutic strategies, including nanomedicine‐based approaches, offer promising avenues for protecting spermatozoa from ferroptosis‐related damage and enhancing reproductive outcomes. Future studies integrating targeted delivery systems and ferroptosis modulators may provide effective interventions for male infertility linked to oxidative stress and iron dysregulation.

## Author Contributions

**Nafiseh Sanei‐Ataabadi:** conceptualization (lead), writing – original draft (lead), writing – review and editing (equal). **Fatemeh Aboutalebi:** conceptualization (equal), writing – original draft (equal). **Kianoush Dormiani:** conceptualization (equal), writing – review and editing (equal). **Mohammad Hossein Nasr‐Esfahani:** conceptualization (lead), writing – review and editing (supporting).

## Conflicts of Interest

The authors declare no conflicts of interest.

## Data Availability

Data sharing not applicable—no new data generated, or the article describes entirely theoretical research.
